# Protocol for a randomised pragmatic policy trial of nicotine products for quitting or long-term substitution in smokers

**DOI:** 10.1186/s12889-015-2366-1

**Published:** 2015-10-06

**Authors:** Doug Fraser, Ron Borland, Coral Gartner

**Affiliations:** School of Public Health, The University of Queensland, Brisbane, Australia; The Cancer Council Victoria, Melbourne, Australia; The University of Queensland Centre for Clinical Research, Brisbane, Australia

**Keywords:** E-cigarettes, Nicotine, Pragmatic trial, Randomized controlled trial, Harm-reduction, Smoking

## Abstract

**Background:**

Smoking is Australia’s leading preventable cause of premature mortality and a major contributor to the national disease burden. If quit rates do not dramatically improve, then smoking will continue to be a major public health issue for decades to come. Harm-reduction approaches using novel nicotine products like e-cigarettes as long term replacements for smoking have the potential to improve quit rates. However, little research has assessed such approaches.

**Methods/Design:**

***Design:*** Three-arm parallel-group pragmatic randomised controlled trial.

***Participants*:** People living in Australia who are at least 18 years old, smoke five or more cigarettes per day and are willing to try a sample of nicotine products.

***Intervention:*** Participants are randomised to receive standard quit advice and medicinal nicotine (Condition A); quit or substitute advice and medicinal nicotine (Condition B); or quit or substitute advice and medicinal nicotine and e-cigarettes (Condition C). Participants choose which (if any) nicotine products to receive to try in a free sample pack followed by a two to three week free supply of their favourite product(s) and the option to purchase more at a discounted price. Follow-up surveys will assess nicotine product use and smoking.

***Primary outcome:*** Continuous abstinence for at least 6 months.

***Target sample size:*** 1600 people (Condition A: 340; Condition B: 630; Condition C: 630) provides at least 80 % power at *p* = 0.05 to detect a 5 % difference in abstinence rates between each condition.

**Discussion:**

This trial will provide data on tobacco harm-reduction approaches and in particular the use of e-cigarettes as a replacement for smoking.

**Trial registration:**

Australian and New Zealand Clinical Trials Registry: ACTRN12612001210864. Date of registration: 15/11/2012.

## Background

Smoking is the largest preventable contributor to Australia’s disease burden [1]. Progress in reducing tobacco-related harm has been slow as quit rates have not changed substantially over the past 30 years [2]. New strategies that encourage more smokers to quit and improve the current modest success rate of quit attempts could greatly reduce the national disease burden [2–5].

**Fig. 1:**
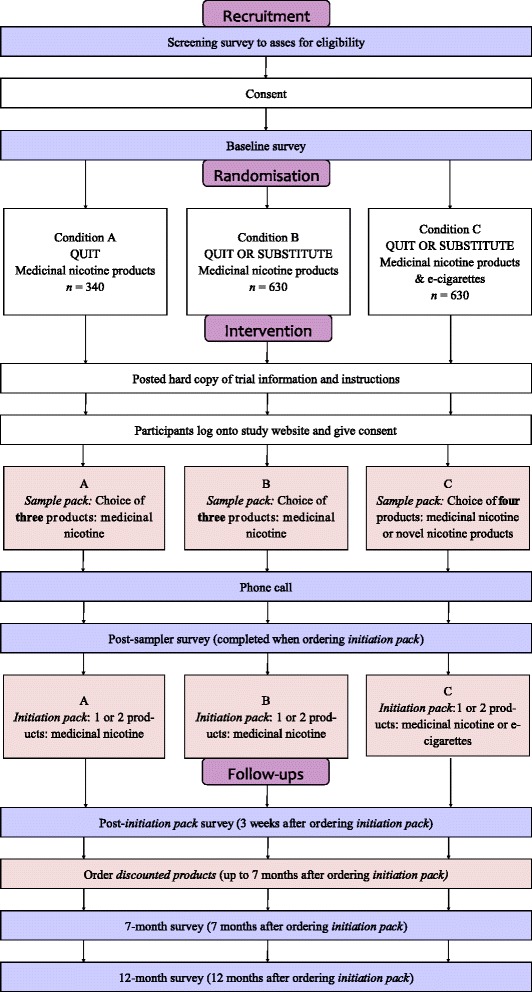
Trial schema showing study design and follow-ups

On an individual basis, substituting cleaner forms of nicotine use for cigarettes would substantially reduce much of the health-related harms of tobacco use [6–8]. If more smokers are able to successfully quit smoking permanently with long-term nicotine use than without it, a policy that encourages either quitting or long-term nicotine substitution could achieve greater population health gains than the current approach of only promoting nicotine abstinence. This harm reduction strategy is advocated by the UK Royal College of Physicians [3, 5] and some tobacco control researchers [7, 9–11].

Harm reduction is one of the three pillars of Australia’s National Tobacco Strategy 2012–2018 [12]. However, there are relatively few harm reduction approaches included in the strategy. Importantly, it identifies the need for research that examines the risks and benefits of alternative nicotine delivery systems, including personal vaporisers or ‘e-cigarettes’. Internationally, the UK government has endorsed nicotine harm reduction and recommends harm-reduction approaches including temporary or long-term use of licensed nicotine-containing products in its NICE Guidance on the topic for smokers who are not able to or do not want to stop using nicotine [13]. The NICE Guidance provides a summary of the evidence in support of this harm reduction approach and also identifies a number of important gaps in the evidence including information on other nicotine delivery systems and the effect of population-level polices and interventions to support harm reduction.

The most promising reduced harm substitutes for cigarettes are medicinal nicotine products, low toxin smokeless tobacco and e-cigarettes. In Australia, medicinal nicotine products come in various forms such as gum, patch, lozenge, inhalator, mouth spray, and dissolvable oral strips. These products are approved as short-term quitting aids [14], are available for sale in general retail outlets and are considered the least harmful method of using nicotine. Clinical trials demonstrate that these products increase quit rates when used as cessation aids [15] but there is less evidence regarding the effect of encouraging their use for long-term substitution in addition to quitting.

Personal vaporisers are a heterogeneous category of nicotine delivery systems. They are all battery-powered devices in which a nicotine solution is heated allowing the user to inhale the resulting aerosol. Earlier models of e-cigarette have relatively small batteries that may be either disposable or rechargeable. Rechargeable versions use replaceable pre-filled cartridges. These earlier models typically resemble combustible cigarettes and are sometimes referred to as ‘cigalikes’. Later models typically consist of a larger rechargeable battery and a replaceable atomiser, mouthpiece and a tank which is filled by the user. Later models often include features that allow users to adjust voltage, wattage, and airflow.

In Australia, nicotine for non-therapeutic use is considered a schedule 7 poison, except when contained in tobacco intended for smoking [14]. Sale, possession and use of a schedule 7 poison without authorisation is illegal in all states and territories [16]. Nicotine in inhaled preparations for human therapeutic use (e.g. smoking cessation) is considered to be a prescription only medicine and must be listed on the Australian Register of Therapeutic Goods before being sold. There are currently no e-cigarettes or nicotine solutions for use in vaporisers listed on the register. Possession and use of non-nicotine e-cigarettes is legal, however sale could contravene tobacco control laws in some states [16].

There is little information on the effectiveness of e-cigarettes for smoking cessation. Cross-sectional studies in countries where e-cigarettes are easy to obtain suggest that many smokers use them as a complete or partial replacement for cigarettes or as a short-medium term quit aid, similar to medicinal nicotine products [17–19]. In a controlled trial, e-cigarettes were found to perform similarly to medicinal nicotine products for smoking cessation [20]. However, a Cochrane review [21] graded the evidence on whether e-cigarettes assist smokers to quit smoking as low due to the small number of high quality trials.

This pragmatic randomised controlled trial will fill an important gap in our understanding of the potential harms and benefits of a low intensity harm reduction intervention: brief advice encouraging long-term use of existing forms of medicinal nicotine (mouth spray, inhalator, gum, lozenge) and e-cigarettes as cigarette substitutes, in addition to advice on short-term use to quit smoking.

### Aim

The primary goal of this study is to rigorously evaluate the effectiveness of two tobacco harm reduction policy options compared to the current smoking cessation policy of recommending short-term use of nicotine products to quit smoking. Specifically, we will determine:

1. If *Quit or Substitute* policies produce more ex-smokers than the current policy of encouraging smokers to *quit with medicinal nicotine* as a short-term quitting aid only;
2. If the proportion of ex-smokers who become long-term users of medicinal nicotine or e-cigarettes increases under the two *Quit or Substitute* policies compared to the current policy.
3. If *Quit or Substitute* policies have detrimental effects on quitting by turning would-be quitters into long-term dual users of medicinal nicotine or e-cigarettes and cigarettes.

### Hypotheses

The proportion of smokers who stop smoking will be greatest under the policy scenario of *Quit or Substitute with medicinal nicotine or e-cigarettes*, followed by *Quit or Substitute with medicinal nicotine* and least in the current policy condition: *Quit with medicinal nicotine*.

We are also interested to see whether the predicted benefit is entirely due to increased use when e-cigarettes are an option, whether the addition of the substitute model adds extra users (i.e., those who want to quit smoking but value the experiences nicotine gives them), and whether there will be any increased rate of cessation among those who do use. This will be explored in subsidiary analyses to the main outcome of overall sustained abstinence.

## Methods/design

### Design

Three-arm parallel-group pragmatic randomised controlled trial. Please see Fig. 1 for study design and follow ups.

### Participants

Adults living in Australia who smoke at least five cigarettes per day and are willing to try a sample of nicotine products.

#### Inclusion criteria

Smokers at least 18 years old, smoke five or more cigarettes per day, are willing to try nicotine products, have internet access, have the ability to read and understand English and are willing to complete online surveys over one year. These criteria are assessed during an online screening questionnaire as part of the initial enrolment process. There is no requirement that the person be planning to quit smoking.

#### Exclusion criteria

Participants are excluded if they are pregnant, breastfeeding, or planning to become pregnant within 12 months, or if they suffer from any of the following conditions: Diabetes mellitus treated with insulin, severe allergies, poorly controlled asthma or other throat or airway disease, stomach ulcer, kidney or liver disease, adrenal gland tumour, overactive thyroid, poorly controlled high blood pressure, a mental health/psychiatric condition that is being treated with regular medication, or stroke, heart attack or severe angina in the previous 2 weeks. Exclusion criteria are assessed by self-report.

### Recruitment

Recruitment is restricted to those jurisdictions that confirmed supplying nicotine e-cigarettes to participants in a clinical trial is permitted (Queensland, New South Wales, Victoria, South Australia, and the Australian Capital Territory). Recruitment is managed by a market research company (I-view Social Research). I-view maintains a national panel of potential participants who are recruited by telephone, face-to-face, word-of-mouth, online, general media and leaflet advertisements. If necessary to meet the sample quota, further targeted advertising in general and social media will allow recruitment of additional participants.

Potential participants receive an email invitation from I-view to participate in the study. The email contains a URL link to an online screening questionnaire which assesses the inclusion and exclusion criteria. Eligible participants are then presented with a brief summary of the study and asked to confirm their consent to participate. Those who consent are directed to the baseline survey and asked to provide their name and contact details.

Upon completing the screening questionnaire and baseline survey, participants are automatically randomised to one of three conditions using block randomisation determined by a computer generated random number sequence. The three conditions represent the current policy situation, and the two potential harm-reduction policy options under examination. Participants are randomised using the ratios and target sample sizes outlined below:

- Condition A: Quit with medicinal nicotine (21 %, *n* = 340)
- Condition B: Quit or substitute with medicinal nicotine (39 %, *n* = 630,)
- Condition C: Quit or substitute with medicinal nicotine and/or e-cigarettes (39 %, *n* = 630)

Automatic checks by I-view on the respondents and manual checks by the researchers will remove ineligible participants. Participants who respond to the screener survey more than once, do not provide a name, valid email address, or other valid contact information (postal address, phone number) are considered ineligible and removed from the study. Likewise, participants who are subsequently discovered to fail the exclusion criteria are ineligible.

### Blinding

Participants are not blinded to the intervention they receive and there is no placebo as all the products are provided as packaged. However, participants only receive information relevant to their allocated condition and are unaware that they have been randomised to a particular condition of study that is offering a different intervention to other participants. More than one participant per household can enrol, however participants residing at the same address as another participant are re-allocated to the same intervention condition as the first enrolling participant from that household to avoid contamination of conditions. Sensitivity analyses will be performed with these participants included and excluded from the dataset. The research team will follow strict protocols for distribution of treatment offers and follow-ups to avoid bias. Outcome measures of cigarette and nicotine product use are collected by the market research company via online surveys. Telephone calls to participants are made by the research team following a standardised script.

### Procedure

After completing the baseline survey online, participants receive more detailed information about the research study in hardcopy mailed to their postal address, including a description of the products they may select from for trial use and instructions for use. A factsheet is included about the harmfulness of nicotine products compared to cigarettes, and possible adverse effects that can be experienced when using these products, and what to do if these occur. Participants in condition A (quit with medicinal nicotine) are encouraged to quit with the assistance of the nicotine products and advised the recommended duration of use is 8–12 weeks. Participants in Conditions B and C (quit or substitute) are encouraged to use the nicotine products as short-term quit aids but to consider using them as a long-term substitute for cigarettes if they are unable to stop using nicotine altogether. The fact sheet in these conditions also includes information on the benefits of quitting or substituting compared to smoking but emphasises complete cessation as the best option from a health perspective. The materials also instruct participants in how to obtain their selected nicotine products for the trial from the study website.

### Experimental products

Two types of nicotine product are offered in the trial, medicinal nicotine and e-cigarettes. The medicinal nicotine products offered to all three conditions include Nicotinell nicotine gum (mint; 2 mg and 4 mg), Nicabate mini lozenges (1.5 mg and 4 mg) with Nicorette Cooldrop lozenges (2 mg and 4 mg) substituted when Nicabate mini lozenges are unavailable, Nicorette Inhalator (15 mg cartridges) and Nicorette Quickmist Mouth Spray (150 mg bottles).

The e-cigarette product - only offered to condition C - is Vype, a disposable e-cigarette available in two nicotine strengths (3.0 and 4.5 %), manufactured by Nicoventures Trading Ltd., with a rechargeable version (Vype Reload) with replaceable cartridges substituted when Vype disposables are unavailable. Vype e-cigarettes are unflavoured and contain nicotine, vegetable glycerin, and water.

Participants receiving e-cigarettes are instructed that the products are only supplied to them for their use while participating in the clinical trial and supply to another person or use beyond the trial dates is not permitted. A wallet-sized card is supplied that confirms the participant has legally been supplied the e-cigarette as part of a clinical trial and provides the research team’s contact details. Participants are also instructed not to use e-cigarettes anywhere smoking is prohibited.

Products are sent via Australia Post or courier depending on cost, delivery timeframes, and regulations covering transport of dangerous goods (i.e., lithium batteries). Participants are required to sign for all parcels and asked to return all unused and used packs of the e-cigarettes via pre-paid pre-addressed postage satchels to the research team for destruction.

### Trial of products

#### Sample pack

Participants can log onto the study website and choose products to try in a *sample pack*. Participants in conditions A and B are able to select up to three of the medicinal nicotine products, while those in condition C will be able to select up to four of the products offered (medicinal nicotine and/or e-cigarettes). They are posted one to two days’ supply of each product selected.

#### Initiation pack

After they have sampled their selected products, they are invited to return to the study website to order one or two products to receive in an *initiation pack* which contains an amount of their selected product(s) that should last approximately two to three weeks. Participants are also allowed to order the *initiation pack* directly rather than first ordering a sample pack, if they already have a clear preference (e.g., from prior experience of using the products)*.* They have up to four months after ordering their *sample pack* to order product(s) for their *initiation pack*. This is to maximise the number likely to use the products, particularly since our recruitment strategy includes participants who have no stated interest in quitting.

#### Discounted products

Participants are able to purchase products from the study website for up to seven months after ordering their *initiation pack*. The price of the medicinal nicotine products is set at approximately 50 % of the mean price of a comparable number of cigarettes or at the lowest price advertised by discount chemists. The prices of products also vary in relation to production costs meaning disposable e-cigarettes are more expensive than simpler products. The aim is to mimic as far as possible a market with price set by a rational government policy using differential taxation that reflects harmfulness. Participants may also purchase products from other retail outlets, however for e-cigarettes, there is no legal supplier in Australia other than the study website.

### Informed consent

After completing the screening questionnaire participants are given a brief description of the study and asked whether they consent to continue. Once they consent and complete the baseline survey, participants are posted the full study information and product descriptions. They can then choose whether to log onto the study website to receive the products. The first time they log onto the website they are again asked to indicate they have understood the research study and consent to continue participating. Participants only receive study products after providing this second consent, however they are categorised as enrolled and included in analyses from the point they were randomised to a condition.

### Withdrawals

Participants can withdraw from the study at any time through an option on the study website or by contacting the research team directly. The can choose whether their data is retained.

### Sample size

In a general population of Australian smokers, 34 % will make a quit attempt over a 7 month period [22]. We expect differential recruitment of those more interested in quitting, and participation in the trial to stimulate more quit attempts than in a natural setting. We assume that approximately 60 % of the sample will take up the products beyond the sample pack, with the proportion of these making a quit attempt similar to that observed in clinical trials. The 12 month abstinence rate in a systematic review of randomised controlled trials of medicinal nicotine for quitting was 12 % [15]. Therefore, we expect an abstinence rate of smokers in condition A of 60 % of 12 %, i.e. 7 %. A sample size of 1600 (N_A_ =340; N_B_ =630; N_C_ =630) will provide greater than 80 % power to detect a 5 % difference in abstinence rates at 12 months (1-tailed test, p < 0.05) between conditions A and B, A and C, and B and C. The unequal allocation between conditions maximises the statistical power for the total sample. Testing between conditions B and C will require a greater sample size than testing between conditions A and B (or A and C) because the difference in proportions to be tested is 12 % vs 17 % rather than 7 % vs 12 %.

A one-tailed test was chosen as the hypotheses are unidirectional because both new policies (Conditions B and C) are departures from the current abstinence only policy and would require a substantial change in current Australian policy. This pragmatic trial is only interested in outcomes in one direction; less conservative and more difficult to implement policies must produce significantly greater quitting than less difficult policies to be given further consideration. If they produce fewer quitters, the policy implications will be the same as if they produced equivalent or inferior results, with no change in *status quo* likely to be supported.

### Data collection

#### Baseline measures

Information collected from each participant includes demographics (date of birth, sex, geographic location, household composition, education); smoking history (age started smoking, nicotine dependence measured with Heaviness of Smoking Index [HSI; time to first cigarette, cigarettes per day] [23], urges to smoke); quit attempts (time since last quit attempt, duration of last attempt); intentions to quit (motivation to quit, current quit plans, perceived difficulty of quitting); prior nicotine product use (experience with medicinal nicotine, e-cigarettes, smokeless tobacco, non-cigarette smoked tobacco; intention to use nicotine products); knowledge of harms of nicotine and smoking (causes of harms from cigarettes and compared to medicinal nicotine, e-cigarettes, and smokeless tobacco); and overall health.

### Follow ups

#### Phone call

Ten days after receiving the *sample pack*, the research team telephone the participant to identify any problems and, if participants are unable to be contacted after three attempts, they are sent an email asking them to confirm they received the sample pack and offer the opportunity to ask questions.

#### Post-sampler survey

When placing their *initiation pack* order, participants complete a brief questionnaire on study website about their experiences with the sample pack and the reasons behind their product choices for the *initiation pack*.

#### Post-initiation pack surveys

Participants are invited by I-view to complete online surveys three weeks, seven months, and one year after ordering their *initiation pack*. They are asked about their product use, smoking, quit attempts, and adverse effects that may be related to using the nicotine products.

Participants who do not order an *initiation pack* are assigned a dummy *initiation pack* order date to allow timing of follow up surveys. If they order a *sample pack* then the date of this order is used, while those who never order a *sample pack* or *initiation pack* are assigned a dummy order date as their baseline survey completion date. Incentives ($30 gift card) are paid to participants for completing each of the final two surveys. I-view Social Research panellists also receive I-view points for completing surveys, which can be exchanged for rewards such as gift cards or charitable donations and entry into a prize draw.

### Primary outcomes

The primary outcome is the percentage in each condition that have:

1. Continuous abstinence (not smoked for at least six months) with or without current/recent medicinal nicotine or e-cigarette use; and
2. Continuous smoking abstinence forat least six months with no medicinal nicotine or e-cigarette use for 3 months or more.

### Secondary outcomes

The secondary outcomes are:

1. Seven-day point prevalence abstinence measures of the above primary outcomes;
2. The proportion of each condition concurrently using medicinal nicotine and cigarettes, the quantity of each consumed, their interest in quitting smoking, and interest in eventually quitting all medicinal nicotine;
3. The proportion of condition C concurrently using e-cigarettes and cigarettes, the quantity of each consumed, their interest in quitting smoking, and interest in eventually quitting all use;
4. The quantity of cigarettes consumed by current smokers as a function of medicinal nicotine or e-cigarette use in each condition, and their interest in quitting smoking;
5. The proportion of each condition that order a product, what product is ordered, the amount used over what period; and
6. The proportions that make a quit attempt and length of all quit attempts made (both from smoking and all nicotine).

### Analytical methods

Participants in the three conditions will be compared on demographic, smoking history and nicotine dependence characteristics to confirm successful randomisation. Participant outcomes will be analysed on an “intention to treat” basis [24], i.e. analysed in the condition to which they were randomly allocated regardless of whether they actually received any of the allocated intervention, subsequently withdrew from the study, or deviated from the protocol. Those lost to follow up will be modelled in various ways including having continued to smoke (the most conservative assumption). This approach gives pragmatic estimates of the benefit of a change in policy under various assumptions, rather than of potential benefit in participants who receive the intervention exactly as planned.

The primary outcomes will be analysed with multivariable logistic regression to determine the odds ratio for each outcome measure for Condition B and Condition C compared to Condition A and for Condition C compared to Condition B. Baseline characteristics (demographic, smoking history and quit intention variables) will be included as covariates to adjust for any baseline differences between the conditions. This will also reduce the impact of any differences between the sample composition and the Australian smoking population. Results will be reported as the odds ratio for quitting smoking and the odds ratio for quitting all nicotine use associated with each of the test policy scenarios compared to the relevant referent categories (Condition A or B).

Subsidiary analyses will explore quitting as a function of trying and of time to relapse, and assess whether relapse is related to cessation of medicinal nicotine/e-cigarettes. Percentages (e.g. percentage who quit) will be calculated with the data weighted to the Australian smoker population using a nationally representative sample (e.g., NDSHS [25]). Unweighted data will be used in the regression analyses.

### Ethical considerations

This research study was approved by the Human Research Ethics Committee of the University of Queensland (Approval Number 2012001117). Consent is obtained during the screening survey and again if participants choose to order nicotine products after receiving the full study information. All data is stored on a secure server only accessible to the researchers.

## Discussion

This study is designed to provide data on the real-world effectiveness of harm reduction policies to inform evidence-based policy decisions. The harm reduction interventions tested in this trial represent a low intensity intervention targeted at a general population of smokers: namely brief advice on long-term substitution with nicotine as a strategy in addition to using it as a short-term quit aid along with access to nicotine e-cigarettes. To have the anticipated effects, the interventions must have greater efficacy among those who use a product, or must attract more smokers to use and subsequently try to quit.

If the trial results indicate that these interventions increase the number of successful quitters in the sample, they will support the inclusion of harm reduction advice in general quit smoking messages and potentially greater access to novel nicotine products, such as e-cigarettes. If we fail to find the anticipated benefit it will be important to determine whether this is because of a failure to increase product trial and use or if there is no greater efficacy as a consequence of adding the substitution advice and an e-cigarette option in addition to standard quit advice. We expect the results of this trial to make a contribution to the debate on whether the current suite of tobacco control strategies should include harm reduction policies, and which nicotine products should be included in such a policy. It will also provide important information as to whether making harm reduced products available along with harm reduction advice results in sufficient numbers take them up for it to affect quit rates.

Our recruitment of participants from a market research company allows a large sample of smokers with varying baseline levels of interest in quitting to be approached in an efficient and low cost way. It is important to recruit smokers with different levels of interest in quitting for this pragmatic trial, as harm reduction messages may be more relevant to non-treatment seeking smokers than those who are already planning to quit smoking. Hence, being motivated to quit smoking is not an eligibility requirement for participation in this trial.

As the intervention is delivered without face-to-face contact with the participants, we selected an earlier generation e-cigarette as these require minimal instructions for use and represent very low risk because the nicotine liquid is contained within a sealed cartridge in the device rather than requiring participants to handle refilling fluids. Furthermore, research suggests that most smokers who switch to e-cigarettes start with a cigalike-style device, suggesting these may initially be more attractive and acceptable to smokers than larger tank style devices [26]. Due to Australian laws that prohibit the supply of nicotine as non-therapeutic products [16], we were restricted in what could be used as the trial product. We selected Vype e-cigarettes for the trial as they met our requirements for quality assurance over the manufacturing of the device and the liquid contained within it, which needs to be of medicinal quality. The e-cigarette market is rapidly evolving with constant improvements in the technology. Therefore, the product tested in this trial will not necessarily be representative of e-cigarettes in general or the most effective e-cigarette on the market in terms of nicotine delivery.

This study relies on self-report for assessing smoking abstinence and use of nicotine products. The majority of data is collected in online surveys delivered via a third party (I-View). Similarly, trial products are ordered via the study website and do not require interaction with the research team unless the participant requires assistance. A follow-up phone call is made early after participants are sent their ordered products to provide an opportunity to ask questions, however smoking outcomes are assessed via the online surveys. The risk of participants inaccurately reporting smoking abstinence due to social desirability bias is low due to the minimal direct contact the participants have with the research team. Because participants in all conditions received an intervention any tendency to please the researchers is likely to be equivalent across conditions. Participants also receive incentive payments for completing the surveys regardless of whether they quit smoking or not.

## Conclusion

Smoking is Australia’s leading preventable cause of premature mortality and a major contributor to the national disease burden. It is also a substantial contributor to the health gap between the least and most disadvantaged members of society, including Indigenous Australians. If quit rates do not dramatically improve, then smoking will continue to be a major public health issue for decades to come. This research will assist national health policy decision making by examining whether tobacco harm reduction strategies could accelerate the current slow rate of decline in smoking prevalence.

## Abbreviations

E-cigarette: Electronic cigarette
NICE: The National Institute for Health and Care Excellence
HSI: Heaviness of Smoking Index
NDSHS: National Drug Strategy Household Survey
